# Genetic Variation of Zinc and Iron Concentration in Normal, Provitamin A and Quality Protein Maize under Stress and Non-Stress Conditions

**DOI:** 10.3390/plants12020270

**Published:** 2023-01-06

**Authors:** Nakai Goredema-Matongera, Thokozile Ndhlela, Angeline van Biljon, Casper N. Kamutando, Jill E. Cairns, Frederic Baudron, Maryke Labuschagne

**Affiliations:** 1 Scientific and Industrial Research and Development Centre, 1574 Alpes Road, Harare 00263, Zimbabwe; 2 International Maize and Wheat Improvement Centre, P.O. Box MP163, Mt Pleasant, Harare 00263, Zimbabwe; 3 Department of Plant Sciences, University of the Free State, P.O. Box 339, Bloemfontein 9300, South Africa; 4 Department of Plant Production Sciences and Technologies, University of Zimbabwe, P.O. Box MP167, Mt Pleasant, Harare 00263, Zimbabwe

**Keywords:** maize, zinc and iron biofortification, micronutrient variability, trait correlations

## Abstract

The negative impacts of zinc (Zn) and iron (Fe) deficiency due to over-reliance on monotonous cereal-based diets are well-documented. Increasing micronutrient densities in maize is currently among top breeders’ priorities. Here, 77 single-cross Zn-enhanced hybrids with normal, provitamin A and quality protein maize genetic backgrounds were evaluated together with seven checks for grain Zn and Fe concentration and agronomic traits under optimum, low nitrogen (N) and managed drought conditions. Results showed a fairly wide variability for grain Zn (10.7–57.8 mg kg^−1^) and Fe (7.1–58.4 mg kg^−1^) concentration amongst the hybrids, across management conditions. Notable differences in Zn concentration were observed between the Zn-enhanced quality protein maize (QPM) (31.5 mg kg^−1^), Zn-enhanced provitamin A maize (28.5 mg kg^−1^), Zn-enhanced normal maize (26.0 mg kg^−1^) and checks (22.9 mg kg^−1^). Although checks showed the lowest micronutrient concentration, they were superior in grain yield (GY) performance, followed by Zn-enhanced normal hybrids. Genotypes grown optimally had higher micronutrient concentrations than those grown under stress. Genotype × environment interaction (G × E) was significant (*p* ≤ 0.01) for GY, grain Zn and Fe concentration, hence micronutrient-rich varieties could be developed for specific environments. Furthermore, correlation between grain Zn and Fe was positive and highly significant (r = 0.97; *p* ≤ 0.01) suggesting the possibility of improving these traits simultaneously. However, the negative correlation between GY and grain Zn (r = −0.44; *p* ≤ 0.01) and between GY and grain Fe concentration (r = −0.43; *p* ≤ 0.01) was significant but of moderate magnitude, suggesting slight dilution effects. Therefore, development of high yielding and micronutrient-dense maize cultivars is possible, which could reduce the highly prevalent micronutrient deficiency in sub-Saharan Africa (SSA).

## 1. Introduction

Grain production for major staple cereals such as maize, wheat and rice has been increasing due to advances in research and technology, with the ultimate goal of meeting the food demand for the continuously growing population [1]. While this improves food self-sufficiency at household and international levels, low densities of micronutrients such as Zn, Fe and vitamin A in these crops remain a global challenge and contribute significantly to micronutrient deficiency [2,3]. Micronutrient deficiency, also referred to as “hidden hunger” is detrimental to health, causing an array of ailments in populations whose diets are characterized by low micronutrient intakes [4,5]. Global statistics estimate that over two billion people suffer from micronutrient deficiency worldwide, although the highest prevalence rate occurs in low income countries where at least half of the population is affected [6,7]. Among the micronutrients, Zn and Fe deficiencies are currently considered as major human health risk factors and cause of stunting, mental illness, anaemia, diarrhoea and reduced ability to do physical work in both children and adults [8,9]. Several studies reported that more than 60% of all infant mortalities are associated with inadequate dietary intake of Zn and Fe [10,11]. All this evidence shows that though required in minute quantities, micronutrient deficiency can cause huge socio-economic losses through reduced national production and increased health care costs [3]. Despite a remarkable reduction in prevalence of various micronutrient deficiencies in most developed countries over the past decades, Zn and Fe deficiencies are still widespread in the developing regions such as in sub-Sahara Africa (SSA) and South Asia. Populations in these regions are worst affected due to overreliance on cereals as a food source, with limited nutritional supplements as well as ignorance of good nutritional practices [12,13]. 

One way of curbing micronutrient deficiencies in developing maize-based regions is to develop micronutrient-dense maize varieties through genetic and agronomic approaches [11,14]. Improving the nutritional quality of maize has always been a priority for breeders, and this strategy complements other micronutrient deficiency mitigation measures, such as dietary diversification, clinical supplementation and industrial fortification [2,15]. To date, several biofortified maize varieties with improved protein quality and high provitamin A and Zn content have been developed and commercialized worldwide, through concerted efforts from both national and international research organizations [2,16]. However, breeding for maize varieties with high levels of grain Zn and Fe concentrations is still lagging behind. To our knowledge, Zn and Fe enhanced maize varieties are still very few, following the recent commercialization of Zn-enhanced ICTA HB-18, ICTA HB-15 and BIO-MZN01 hybrids in Guatemala and Colombia, respectively [17].

Breeding for genotypes with high levels of grain Zn and Fe concentration depends on the existence of sufficient genetic variability for such traits in germplasm. Significant genetic variation in grain Zn and Fe concentration has been reported [4,18]. In addition, some studies reported positive association between grain Zn and Fe concentration and between grain yield and micronutrient concentration, suggesting the possibility of simultaneous improvement of such traits [4,19]. Therefore, knowledge of the nature of phenotypic correlations between traits is important in plant breeding and facilitates indirect selection of such traits. Moreover, the ability to accumulate significant concentrations of Zn and Fe in edible crop parts differs among maize genotypes, and sometimes depends on the root microbiome and soil physicochemical properties such as total soil Zn and Fe, extractable Zn and Fe, soil pH, organic matter and moisture content [20,21]. The soil must have sufficient plant-available Zn and Fe concentration for absorption by the maize roots. Among the soil chemical factors, soil pH plays the most important role in Zn and Fe solubility in soil solution. Previous studies reported about 30 to 50% decrease in Zn concentration in soil solution for each unit increase in pH [7,14]. Hence, a slight increase of soil pH from 5.5 can increase the risk of Zn deficiency in maize. Other studies reported sufficiently high Zn^2+^ in soil solution when pH is maintained at 5.0 [14]. In addition, soil organic matter increases solubility and transport of Zn to plant roots [14]. Low grain Zn and Fe concentrations in maize can be caused by moisture stress. Transport of these micronutrients to the root surface in soils occurs predominantly via diffusion, and hence adequate soil moisture is critical for providing suitable medium for that process [20].

Whilst several studies have focused on assessing the genetic variability of maize inbred lines and hybrids [1,4,13], very few studies have focused on how maize genotypes with different nutritional background, soil physicochemical properties, and crop management conditions can affect the accumulation of Zn and Fe in maize grain. This information is very useful in optimizing both agronomic and genetic biofortification as interventions to combat micronutrient deficiencies in affected regions. Therefore, the objectives of this study were: (i) to determine the variation of total soil extractable Zn and Fe and other physicochemical properties in maize experimental sites, (ii) to determine the variation of grain Zn and Fe concentration and other agronomic traits and its association with soil extractable Zn and Fe, (iii) to examine correlation between grain Zn and Fe and agronomic traits using Zn-enhanced normal (NML), provitamin A (ProA) and QPM genotypes grown under optimum, low N and managed drought conditions.

## 2. Results

### 2.1. Soil Chemistry

The summary of the descriptive statistical data for soil physiochemical properties analysed in soil samples that were taken in four experimental stations across two years is presented in Table 1. All the sites showed highly significant differences (*p* ≤ 0.05) for all the soil physicochemical properties except for inorganic carbon (IC). Soil pH ranged from moderately acidic (5.70–5.80), slightly acidic (6.10–6.40) to slightly alkaline (7.60) across sites and years (Table 1). The lowest and highest pH was observed at ART farm and Chisumbanje, respectively. Total carbon (TC) was the highest at CIMMYT under optimum management conditions and lowest at DR&SS under low N conditions. Nitrogen content (TN) was similar for all sites except for soil from DR&SS (low N site), which had <5% N content (Table 1). A similar trend was also observed for soil organic carbon content (SOC), which is the primary component of soil organic matter. Results showed significant differences (*p* ≤ 0.05) in available P (Olsen P extractable) content, with the highest value observed in soils from ART farm (25.9 mg kg^−1^) and lowest at CIMMYT (10.4 mg kg^−1^). Cation exchange capacity (CEC), a useful indicator of soil fertility ranged from 14.2–28.1 and 12.1–23.4 meq 100 g^−1^ in 2018/19 and 2019/20, respectively (Table 1). Extractable Zn concentration was lower than for Fe in all sites. Soils from ART farm had the highest soil Zn concentration with 5.10 and 5.70 mg kg^−1^ in 2018/19 and 2019/20, respectively, while Chisumbanje had the lowest values across the two years. In terms of soil texture, the highest clay content was observed in Chisumbanje, whilst DR&SS had proportionally higher sand content than other sites.

### 2.2. Agronomic and Micronutrient Performance of Zn-Enhanced Hybrids

**Figure 1:**
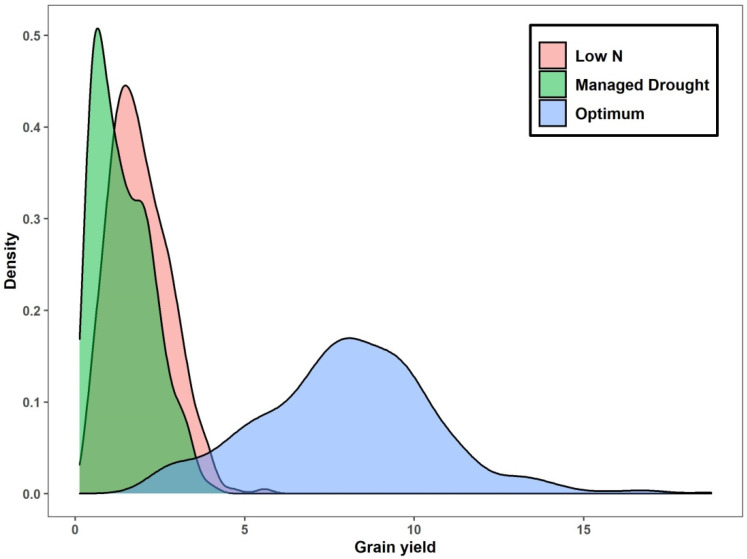
Density plot for grain yield (t ha^−1^) performance of test hybrids under optimum, low nitrogen and managed drought conditions. The area under the curve represents the probability of getting hybrids with certain grain yield performance along the x-axis.

The means for days to mid-anthesis (AD) for the hybrids did not differ much among the three management conditions (Table 2). However, the means for ASI showed notable differences. Under optimum management, means for anthesis-silking-interval (ASI) were ≤2.00 days across sites and years, while slightly higher mean values were observed under low N and managed drought conditions. Plants were shortest in E8 (161 cm) under managed drought conditions, and tallest in E2 (245 cm) under optimum conditions. Similarly, the mean grain yield performance of hybrids was highest in E6 (9.30 t ha^−1^) under optimum conditions, and lowest in E8 (1.10 t ha^−1^) under managed drought conditions. Combined analysis for GY indicated that most hybrids performed better under optimum than stress conditions (Figure 1).

The means for grain Zn concentration were higher than for Fe in most sites. The highest mean for grain Zn was observed in E1 (30.7 mg kg^−1^) followed by E6 (30.4 mg kg^−1^) which were optimum sites, whereas stress induced sites E3 and E8 had the lowest grain Zn means of 24.8 mg kg^−1^ and 25.1 mg kg^−1^, respectively. Grain Fe concentration was lowest in E3 (20.4 mg kg−1) and highest in E6 (30.8 mg kg^−1^). Genetic variability for grain Zn was highest in E6 (15.6–57.8 mg kg^−1^), followed by E2 (16.4–49.2 mg kg^−1^). For grain Fe concentration, highest and lowest genetic variability was observed in E5 (12.6–58.4 mg kg^−1^) and E3 (7.81–34.9 mg kg^−1^). Narrower genetic variability for grain Zn and Fe concentration was observed in E3, E4, E7 and E8 (drought and low N) than under optimum conditions (Table 2).

### 2.3. Genotypic Variance, G × E Interaction and Heritability

Highly significant differences for δ^2^_g_ were evident for all the six traits, except for AD (E2) and ASI (E2 and E6) as shown in Table 3. Among the agronomic traits, plant height (PH) had the highest δ^2^_g_ in most individual experimental sites, with the highest value in E2. A similar trend was observed across sites. GY also showed highly significant δ^2^_g_ in both individual locations and across environments, with the highest value at E2 (Table 3). The results indicate that in all the sites across years, δ^2^_g_ for GY was higher at optimum sites than at stress-induced sites. The δ^2^_gy_ interactions were not significant for all the agronomic traits, whereas for δ^2^_ge_ interactions, only AD and GY were significant. Comparing the two agronomic traits, δ^2^_ge_ for AD was significant but with a small magnitude, whilst for GY, δ^2^_ge_ was highly significant (Table 4). Although δ^2^_gye_ interactions were significant for all the agronomic traits, its significance was less than for genotypic variance of these traits.

For micronutrient concentration, the δ^2^_g_ for grain Zn and Fe concentration was highly significant (*p* ≤ 0.01) in all individual experimental sites (Table 3). Grain Zn concentration had the highest genotypic variance in E1 (CIMMYT 2018/19), and lowest in E7 (DR&SS 2019/20). For grain Fe concentration, the highest δ^2^_g_ was observed in E5 (CIMMYT 2019/20), whereas E4 (Chisumbanje 2018/19) had the lowest genotypic variance. Similar to observations on GY, δ^2^_g_ for grain micronutrient concentration was higher under optimum sites than under managed drought or low N conditions. While δ^2^_g_ was significant across sites using pooled data, δ^2^_gy_ interactions were not significant for both grain Zn and Fe concentrations (Table 4). However, both δ^2^_ge_ and δ^2^_gye_ interactions were highly significant (*p* ≤ 0.01). Apparently, δ^2^_ge_ interactions for the micronutrients were higher than δ^2^_g_.

Most of the phenotypic traits were highly heritable (H^2^ > 60%) at individual experimental sites except for AD (E2) and ASI in several sites (Table 3). In stress and non-stress environments, PH, GY, grain Zn and Fe concentrations had the highest heritability. Grain yield was more heritable under optimum than stress sites. In contrast, heritability estimates in stress and non-stress environments were similar for grain micronutrients. Compared to agronomic traits, higher heritability was observed for grain micronutrients (Table 3). In the combined analysis, heritability ranged from 56–89%, with GY having the highest heritability, followed by PH (Table 4). Comparing the two micronutrients, grain Zn concentration was more heritable than grain Fe concentration across sites and years.

### 2.4. Grain Yield and Micronutrient Performance by Nutritional Type

**Figure 2:**
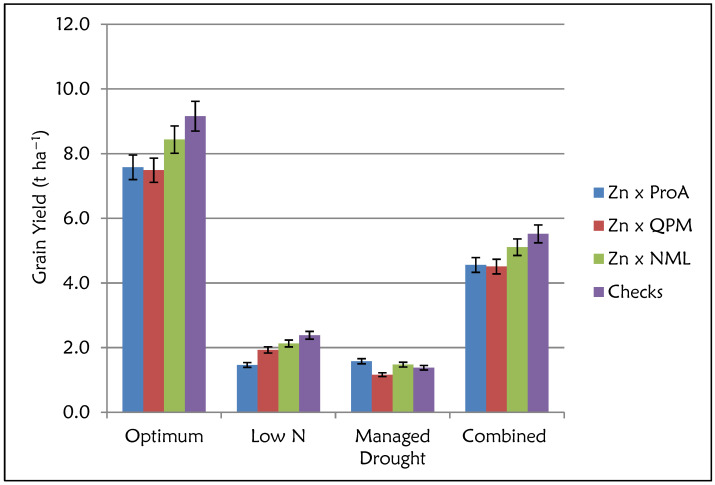
Grain yield performance (t ha^−1^) for the Zn-enhanced normal (Zn × NML), provitamin A (Zn × ProA) and QPM (Zn × QPM) testcross hybrids and checks evaluated under stress and non-stress environments.

**Figure 3:**
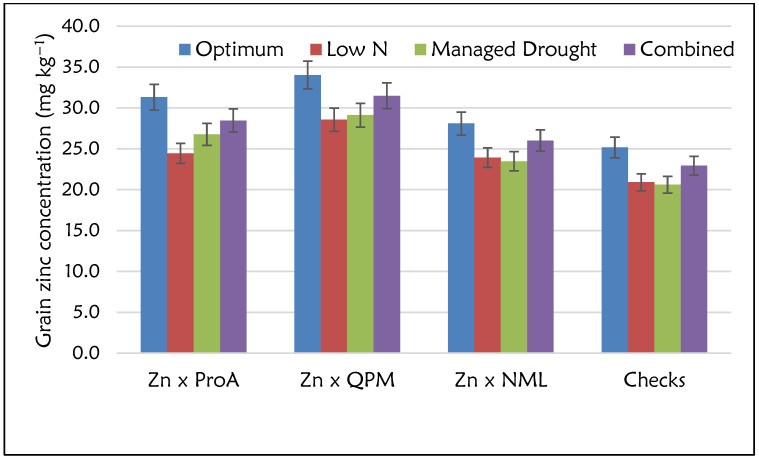
Grain zinc concentration (mg kg^−1^) for the Zn-enhanced normal (Zn × NML), provitamin A (Zn × ProA) and QPM (Zn × QPM) testcross hybrids and checks evaluated under stress and non-stress environments.

**Figure 4:**
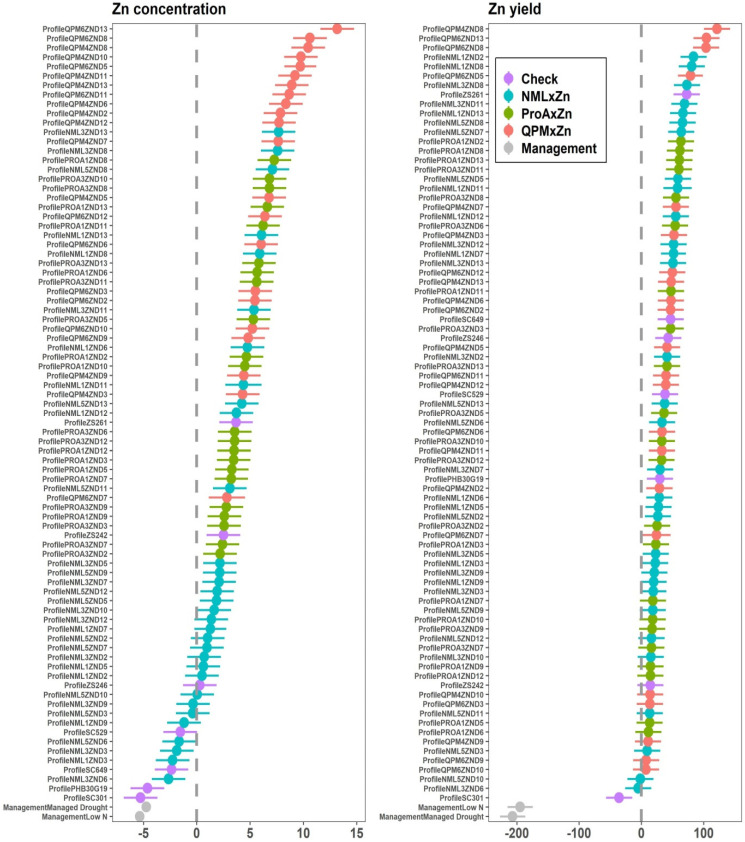
Nutritional profile showing Zn concentration versus Zn yield of testcross hybrids and checks grown under low N and managed drought conditions where optimum management was the reference.

**Figure 5:**
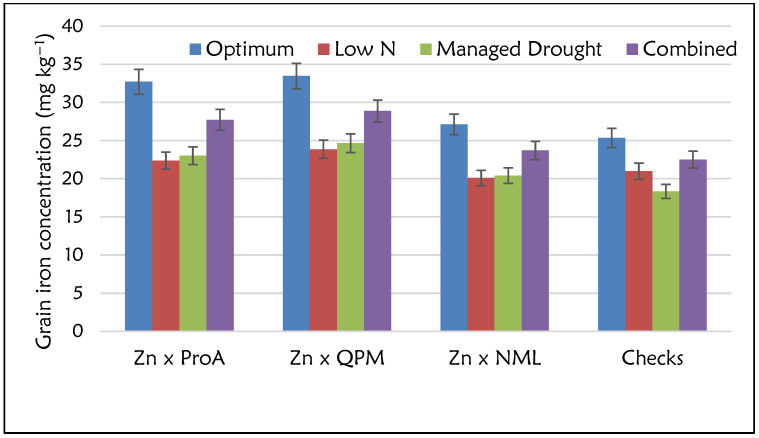
Grain iron concentration (mg kg^−1^) for the Zn-enhanced normal (Zn × NML), provitamin A (Zn × ProA) and QPM (Zn × QPM) testcross hybrids and checks evaluated under stress and non-stress environments.

The Analysis of Variance (ANOVA) showed significant differences (*p* ≤ 0.05) for GY performance among the four nutritional groups: Zn × NML, Zn × ProA, Zn × QPM and checks (ANOVA table not shown). The effects of management and management × nutritional group interactions were significant (*p* ≤ 0.05). For all the nutritional groups, GY performance was highest under optimum conditions and lowest under managed drought conditions. Commercial checks had the highest GY under optimum (8.40 t ha^−1^) and low N (2.40 t ha^−1^) conditions and across sites (Figure 2). However, the GY performance of the Zn × NML category was comparable to that of the checks. Despite the lower GY performance, Zn × ProA and Zn × QPM hybrids yielded at least 7.00 t ha^−1^ under optimum conditions. GY performance of these two nutritional groups was comparable for most management conditions. However, Zn × ProA hybrids had the highest GY performance under managed drought conditions, although there were no large differences among the four nutritional groups. ANOVA for grain Zn and Fe concentration showed highly significant differences (*p* ≤ 0.05) for the management and nutritional groups (data not shown). However, management × nutritional type interaction was non-significant for grain Zn concentration, whereas high significance was observed for Fe. For all the nutritional groups, higher grain Zn concentrations were observed under optimum than stress conditions (Figure 3). Zn × QPM hybrids accumulated the highest grain Zn concentration (34.0 mg kg^−1^) across all management levels (Figure 3), followed by Zn × ProA hybrids (31.3 mg kg^−1^). Commercial checks had the lowest grain Zn concentration (25.2 mg kg^−1^). The general observation across sites in terms of grain Zn accumulation was Zn × QPM > Zn × ProA > Zn × NML > check hybrids (Figure 4). The Zn × QPM and Zn × ProA hybrids accumulated higher grain Fe concentration than Zn × NML and checks (Figure 5). However, cross-over interactions were observed between nutritional groups and management levels for grain Fe concentration.

### 2.5. Correlations between Agronomic and Micronutrient Traits

Grain Zn and Fe concentration showed positive and significant correlations (Table 5) at all individual sites (E1 = 0.75, E2 = 0.72, E3 = 0.52, E4 = 0.67, E5 = 0.56, E6 = 0.70, E7 = 0.54 and E8 = 0.51; *p* ≤ 0.01). Across site correlation analysis, a similar trend was observed (r = 0.97; *p* ≤ 0.01). The association between GY and grain Zn concentration was negative and non-significant in all environments, except for two sites (E7 and E8), but in all cases the magnitude was small. However, across site analysis revealed that GY and grain Zn concentration were moderately and significantly correlated (r = −0.44; *p* ≤ 0.01). In contrast, no trend was observed for correlation between GY and grain Fe concentration. However, the correlation of these traits was moderate, negative and significant (r = −0.43; *p* ≤ 0.01) across sites (Table 5). The association between grain Zn concentration and either AD or ASI was not significant for all sites except for AD in E2. In contrast, across site analysis indicated positive and significant association between these traits. Highly positive and significant correlation was observed between PH and GY in individual sites and across sites (E1 = 0.58, E2 = 0.66, E3 = 0.57, E4 = 0.28, E5 = 0.74, E6 = 0.58, E7 = 0.61 and E8 = 0.83; Pooled = 0.99; *p* ≤ 0.01). AD was positively associated with GY at all optimum sites except for E6, while high and significant negative correlation was observed under stress conditions (E3, E4, E7 and E8). The association between ASI and GY was negative and stronger under stress than non-stress environments.

## 3. Discussion

The current study aimed to, first, determine the soil extractable Zn and Fe concentration in major maize experimental sites in Zimbabwe, second, evaluate the genetic variability of grain Zn and Fe concentration in newly developed Zn-enhanced normal, provitamin A and QPM testcrosses and link this with soil physicochemical properties and crop management; third, examine correlation between grain Zn and Fe concentration and agronomic traits, in an effort to develop high yielding Zn-enhanced cultivars with substantial grain Fe concentration.

Moderate but significant diversity at the experimental sites in terms of soil extractable Zn and Fe concentration, pH, total soil carbon, soil organic carbon and texture was observed. All these soil properties, including the root microbiome are critical in determining the extent of soil Zn and Fe solubility and subsequent accumulation in edible crop parts [14,22]. The soil pH for sites ranged from slightly acidic to slightly alkaline where maize normally grows optimally [23,24]. Having soil pH of such range implies that the pH had minimum inhibitory effects to grain Zn and Fe accumulation, considering that bioavailability of these micronutrients is greatly affected under extremes of both acidity and alkalinity [25]. Experimental sites differed in terms of soil extractable Zn and Fe concentration, with ART farm and CIMMYT having the highest soil extractable Zn and Fe concentration, respectively, while Chisumbanje had the lowest concentration for the two micronutrients. Although experimental sites differed in terms of soil extractable Zn and Fe, all the sites had sufficient concentrations of these micronutrients required to sustain maize growth and development based on critical limits of 1.00 and 5.00 mg kg^−1^ for soil Zn and Fe, respectively [26]. Previous studies have demonstrated that grain Zn and Fe concentration in maize sharply increases with an increase in soil extractable Zn and Fe concentration [7,27]. Whilst genotypic differences observed in the present study for grain Zn and Fe concentration between sites could have been attributed to differences in soil extractable Zn and Fe concentration, the impact was small. This is because the low N sites (E3 and E7) had comparably higher soil extractable micronutrients but lower means for grain Zn and Fe concentration.

Differences in means and extent of genetic variability for grain Zn and Fe concentration observed among the three crop management practices suggest high significance of crop management in determining grain micronutrient concentration. It is well documented that nutrient uptake decreases drastically under moisture stress conditions [28,29] and this could explain the low grain Zn and Fe concentration at all the managed drought sites. In addition, the combination of both optimal N fertilizer rates applied and higher inherent soil N content at CIMMYT 2018/19 (E1) and ART farm 2019/20 (E5) could be responsible for higher grain Zn and Fe in those sites as compared to low N sites. Previously, a 14.6–26.9% increase in grain Zn concentration in maize compared to trials with no N application was reported [30]. In the present study, optimum crop management increased the grain Zn concentration by 21.5% as compared to the average of all low N sites. Similar findings have also been reported by other authors [31,32]. Contrary to this, a different study [33] reported negative correlations between N fertilizer application and grain Zn concentration. As expected, higher N sites in the present study were associated with higher soil organic carbon, a major component of soil organic matter [34]. Direct and indirect influence of soil organic matter on soil micronutrient availability and uptake by plants was reported in different studies [35,36]. All these results demonstrate that the accumulation of micronutrients in maize grain is highly dependent of several environmental factors [14]. Although results from this study demonstrate significant differences in terms of soil physicochemical properties that might influence grain Zn and Fe concentration in maize, the experimental sites used were not fully representative of target environments, since most smallholder farmers in Zimbabwe grow maize in highly acidic sandy soils with very low organic matter [37]. Future studies should consider conducting such experiments in smallholder farms that are often characterized by low productivity potential. In addition to that, future studies should explore the correlation between extractable Zn and Fe concentration in soil solution and the micronutrient concentration accumulated in maize grains.

The mean performance of genotypes in both individual and across environments shows that sufficient genetic variability for both agronomic and micronutrient traits exist. This suggests that improvement of grain micronutrients and agronomic traits in locally adapted normal, provitamin A and QPM germplasm is possible. Genotypic variability in the gene pool available to the breeder is crucial, as this facilitates selection of genotypes with superior performance in traits in question [38]. Genotypic variances observed for most traits in the present study were highly significant coupled with high heritability, indicating great potential to improve such traits (Table 3). Plant height had the highest genotypic variance in almost all sites, indicating strong genetic control of this trait and minimum environmental influence [39]. The traits phenotyped in this study are quantitative and are influenced by several genes interacting with various environmental factors, and this justifies highly significant δ^2^_ge_ for these traits. G × E interaction reduces the broad-sense heritability across sites and this ultimately reduces the selection accuracy [40]. Comparing the two micronutrients, grain Fe concentration had higher δ^2^_ge_ than grain Zn concentration and this indicates stronger influence of the environment for Fe than for Zn. However, δ^2^_gy_ was non-significant, implying that growing seasons were similar across the years. In contrast, δ^2^_gye_ was highly significant and of greater magnitude compared to δ^2^_g_, for all traits except for grain Zn and Fe concentration. This shows that the environment highly influenced agronomic traits compared to micronutrient concentration. A similar low contribution of the environment to grain Fe and Zn concentration was reported [41,42] in pearl millets. Contrary to these findings, another study [19] reported a bigger contribution of the environment to the total micronutrient variation in sorghum. Comparing the two micronutrients, δ^2^_gye_ for grain Fe was higher than for grain Zn concentrations and these results corroborate previous findings [19] in sorghum, but are contrary to reports in maize [43]. Greater proportion of genotype × environment × year effect for grain Fe suggests that this micronutrient is more sensitive to environmental changes than grain Zn concentration. This further supports the well-documented evidence that the accumulation of grain micronutrients is highly influenced by several factors including soil moisture, pH, soil type and fertility, genotype and interactions of among nutrients in soil [14,43]. The presence of large genotypic variance and moderate G × E interactions for grain Zn and Fe concentration suggests the possibility to increase the mineral concentration through both modern and conventional breeding approaches.

The optimum environments were more favourable to most Zn-enhanced genotypes compared to stress environments. Both drought and low N stress increases kernel abortion, delays silking, reduces the number of ears per plant, which all have negative impacts to grain yield [44]. Furthermore, reduced soil moisture could have contributed to the low micronutrient absorption and uptake. Several studies have demonstrated that grain Zn concentration in maize [7,30], and wheat [45] increases with N supply. Therefore, the N fertilizer limitation in DR&SS site might have decreased grain Zn concentration. Evidence on whether N supply has direct influence on grain Fe accumulation in maize is still limited, although few studies have reported significant improvement of grain Fe with N application in cowpea [46].

The nutritional backgrounds (types) of the evaluated hybrids were also significant in determining grain yield and micronutrient concentration. The highest grain yield observed in non-biofortified commercial checks, followed by Zn × NML hybrids concur with several studies reporting dilution effects [4,17]. However, the differences in grain yield performance between Zn-enhanced hybrids and checks were not large and this suggests the possibility of developing high yielding and nutritionally superior maize genotypes. In terms of micronutrient performance, the QPM-based hybrids had comparably better performance than other nutritional backgrounds. The superiority of QPM germplasm for grain Zn concentration has been reported by several authors [1,8,13,47]. However, high grain Zn in normal maize has also been reported [48] and this is quite encouraging to breeders. The positive and highly significant correlation between grain Zn and Fe concentration suggests that the genes governing accumulation of these micronutrients could be linked, implying that simultaneous improvement is possible [4]. Correlation between grain yield and either grain Zn or Fe concentration was negative and moderate, although it was highly significant. While other studies have reported similar findings [4], there have also been contrasting results [13,49]. Based on the results, it is apparent that the dilution effects were significant but only to a limited extent. This shows that genotypes with moderate to high yield potential coupled with high levels of grain micronutrients can be developed. Application of foliar Zn-containing fertilizers can also increase the grain Zn concentration in case of high yielding cultivars with moderate ability to accumulate grain Zn in soil [49].

## 4. Materials and Methods

### 4.1. Plant Materials

Seventy-seven Zn-enhanced normal, provitamin A and QPM testcross hybrids were developed by crossing 11 Zn donors derived from CIMMYT-Mexico and the International Institute of Tropical Agriculture (IITA) in West Africa and 7 testers of normal, provitamin A and QPM backgrounds. Testers were developed by CIMMYT in Southern Africa. Testcrossing was done during the 2017/18 cropping season at CIMMYT’s Harare Station (Latitude = 17°43′ S; Longitude = (31°05′ E); Altitude = 1480 masl; Average annual rainfall = 850 mm). Planting followed an 11 × 7 North Carolina II design, with Zn donors used as males while normal, provitamin A and QPM line testers were used as females. The testers were locally adapted tropical germplasm that has been widely used in evaluating newly developed inbred lines. Baseline Zn and Fe concentrations for the potential parental lines were measured (Table 6) and final selections were based on considerable micronutrient densities and tolerance to biotic and abiotic stresses such as drought and disease (maize streak virus and grey leaf spot). In addition, the provitamin A and QPM testers were previously analysed and selected for high levels of β-carotene and tryptophan content. All the generated 77 testcross hybrids had enough seed quantities to be evaluated in trials together with seven commercial checks.

### 4.2. Experimental Sites

The study was conducted in eight environments in Zimbabwe (Table 7), during the 2018/19 and 2019/20 cropping seasons. These sites are widely used for evaluating agronomic performance of breeding materials under development. The testcross hybrids were grown under two optimum, one low N and one managed drought conditions for two years. Both optimum and low N trials were planted in the main (summer) cropping season, while the managed drought trials were grown under irrigation during the winter, rain-free period. Maize plants were exposed to drought stress by withholding irrigation two weeks before flowering up to 21 days after flowering, so that drought stress would coincide with flowering, which is the most sensitive growth stage. Similarly, the low N trials were grown in low N screening sites that were developed by continuously depleting N to less than 7 ppm, which is estimated to cause 30% maize yield reduction [50].

In Zimbabwe, agro-ecological zones, also known as natural farming regions (NFRI–V), are defined based on the annual rainfall amount received in a particular area, and soil quality and type of vegetation, among other factors [51]. NFR1 receives the highest annual rainfall amount of >1000 mm and NFRV receives the least rainfall amount of <450 mm^7^. A representation of high and low potential environments with contrasting agro-ecologies was included in this study (Table 7).

### 4.3. Trial Layout, Management and Data Collection

The 84 genotypes were laid out in the field using an alpha (0.1) lattice design with two replications. Briefly, each replicate accommodated 12 incomplete blocks with a block size of 7 genotypes. Genotypes were planted in single row plots of 4 m long with 17 planting stations. Two seeds were sown in each planting hole and thinned two weeks after emergence to achieve a uniform crop stand. Spacing was 0.75 m (inter-row) and 0.25 m (in-row) to achieve a plant density of 53,000 ha^–1^. A total of four border rows were planted on each side of the main experimental plot.

Standard agronomic and cultural practices were applied at all sites during crop growth and development. Weed control was done using herbicides and in some cases hand weeding was applied. Supplementary irrigation was applied when necessary in all the trials during the early vegetative stages. Two random plants from each plot were selfed to produce seed for the micronutrient analysis and all other plants were allowed to open-pollinate for phenotypic traits data collection, primarily for grain yield (GY), days to mid-anthesis (AD), anthesis-silking-interval (ASI) and plant height (PH). GY recorded using plants in the net plot area as the two border plants close to the alley were discarded. AD was recorded as number of days from planting to when 50% of the plants had tassels shedding pollen. Plant height (PH) was recorded using a laser distance meter as the average of all plants in the plot. Lastly, ASI was determined as the difference between days to silking and anthesis.

### 4.4. Soil Micronutrient Analysis

Soil analysis was done to determine the soil micronutrient concentration and other physicochemical properties for each experimental site used. Soil samples were taken using a 20 cm hand operated auger in an M-shaped pattern after scraping away surface litter [52]. The edges of the field were excluded from sampling to avoid contamination from residual fertilizers, since bags are normally placed at the periphery of the field during application. A total of 15 subsamples were collected at each experimental site to make a composite soil sample for analysis. Soil samples were sent to Rothamsted Research Soil Science Laboratory, United Kingdom. The primary micronutrients, including Zn, Fe, magnesium (Mg), and copper (Cu) were simultaneously extracted using the Mehlich 3 method [53]. Extractable micronutrients were determined by an inductively coupled plasma-optical emission spectrophotometer (ICP-OES; model: ICAP 6000 series, Thermo Fisher Scientific, Dreieich, Germany). The conventional Olsen P method [54] was used for determining available P. Extraction of exchangeable metal cations such as calcium (Ca), potassium (K), Mg, and sodium (Na) was done using ammonium chloride [55]. Total nitrogen (TN) and total carbon (TC) were determined using the Dumas combustion procedure using the Leco CNS 2000 analyzer [56]. Inorganic carbon (IC) was determined using finely ground (0.15 mm) soils in the Primacs SNC analyser (Skalar Inc., Buford, Georgia). Soil pH was measured in a 1:2.5 soil/water mixture using an electrode pH meter.

### 4.5. Grain Micronutrient Analysis

Ears from the selfed maize plants were manually harvested and shelled to avoid any metal contamination from mechanical harvesting. Grain of all selfed plants in a plot was bulked to form a 100 g composite sample. Fifty kernels per sample were ground using a Foss mini flour industrial maize grinder. Milling time was 60 s at 15 Hz. The flour was sieved through a 1 mm stainless steel sieve and stored at 4 °C in screw-top polycarbonate vials. Two grams of flour was dry-ashed in porcelain crucibles, placed in a muffle furnace at 550 °C for 3 h and digested using concentrated HNO_3_ acid [57]. Grain micronutrient concentration (Zn and Fe) was determined using the atomic absorption spectrometry (AAS) [58].

### 4.6. Statistical Analysis

Analysis of variance (ANOVA) for the phenotypic traits of testcross hybrids across environments was carried out using the Best Linear Unbiased Prediction (BLUP) approach in Multi-Environmental Trial Analysis (META-R) v6.0 [59]. The variance components (δ^2^_g_, δ^2^_gy_, δ^2^_ge_, δ^2^_gyl_ and δ^2^_ε_) were estimated in the linear mixed model, where the environment and year were considered as fixed factors, while genotypes, replicates, blocks and genotype interactions with year and environment were considered as random effects. Grain yield was adjusted to 12.5% moisture content before analysis. Pooling data for combined analysis was done after conducting a Bartlett’s test for testing for homogeneity of variances between environments and years [60]. GenStat software 17th edition [61] was also used for generating descriptive statistics.

Genotypic correlations among micronutrient and agronomic traits were performed using BLUPs of individual and across sites in META-R. The combined analysis, taking into account the δ^2^_gye_ effect was performed using the following model:

Y_jklmn_ = µ + Y_j_ + E_k_ + YE_jk_ + r_jkl_ + B_jklm_ + G_n_ + (YG)_jn_ + (EG)_kn_ + (YEG)_jkn_ + ε_jklmn_

where µ is the grand mean; Y_j_ is the fixed effect of year j; E_k_ is the fixed effect of environment k; YE_jk_ is the fixed effect of interaction between year j and environment k; r_jkl_ is the random effect of replication l in environment k and year j; B_jklm_ is the random effect of block m nested in replication l in environment k and year j; G_n_ is the random effect of genotype n; (YG)_jn_ is the random effect of the interaction between genotype n and year j; (EG)_kn_ is the random effect of the interaction between genotype n and environment k; (YEG)_jkn_ is the random effect of the interaction effect of the genotype n in environment k and year j; ε_jklmn_ is the residual variance.

Broad sense heritability (H^2^) for traits was estimated in META-R using the following formula:

$$H^{2}=\frac{\delta_{g}^{2}}{\delta_{g\;}^{2}+\delta_{\varepsilon /r\;}^{2}}\;$$

Where δ^2^_g_ is the genotypic variance, r and δ^2^_ε_ denotes the number of replicates and residual variance, respectively.

## 5. Conclusions

The current study showed significant differences (genotypic and between nutritional type) in terms of both micronutrient and agronomic traits under optimum, low N and managed drought conditions. Experimental sites with high soil N, total carbon and organic matter had the highest grain Zn and Fe concentration and therefore agronomic biofortification could be useful in increasing these micronutrients in maize grain. Further studies using large populations are required for validation of the genotypic differences in micronutrient densities between normal, provitamin A and QPM germplasm. Considering limited differences in soil physicochemical properties in experimental sites observed in the present study, further studies should explore differences in smallholder farms, which are normally characterized by low clay to sand ratio. High and significant correlation between grain Zn and Fe concentration indicate the possibility of simultaneous improvement of these traits, which is quite cost-effective. Despite showing lower grain yields, micronutrient content of Zn-enhanced hybrids was higher than the control. Although, grain yield was negatively correlated with both grain micronutrients, the magnitude of the correlation coefficient was moderate and this gives hope in developing high yielding and micronutrient-dense maize cultivars, which could reduce the highly prevalent micronutrient deficiency in SSA.

## Figures and Tables

**Table 1:** Means for extractable soil physicochemical properties of different experimental sites.

				2019									
Site	Management	pH	TC (%)	IC (%)	TN (%)	SOC (%)	Olsen P mg kg^−1^	CEC meq^−100 g^	Zn mg kg^−1^	Fe mg kg^−1^	% Clay	% Silt	% Sand
ART farm	Optimum	5.80	1.40	0.02	0.15	1.30	25.9	14.2	5.10	57.8	39.5	49.5	11.0
CIMMYT	Optimum	6.40	2.20	0.01	0.20	2.20	10.4	24.5	2.70	76.1	40.5	40.5	19.0
DR&SS	Low N	6.10	0.80	0.02	0.04	0.70	17.8	16.4	4.70	56.1	52.0	26.5	21.5
Chisumbanje	Managed drought	7.60	1.70	0.03	0.15	1.70	15.7	28.1	1.90	41.1	73.0	26.0	1.00
^†^ F-test		*	**		**	**	**	**	**	**	**	**	**
				2020									
ART farm	Optimum	5.70	1.40	0.02	0.15	1.40	23.4	13.2	5.70	52.3	37.5	48.5	14.0
CIMMYT	Optimum	6.30	2.30	0.01	0.18	2.30	12.1	25.8	2.70	77.9	41.5	39.5	19.0
DR&SS	Low N	6.20	0.60	0.02	0.05	0.60	18.5	14.9	4.80	57.6	51.5	26.0	22.5
Chisumbanje	Managed drought	7.60	1.80	0.02	0.17	1.70	16.0	29.1	2.00	39.1	72.5	25.0	2.50
^†^ F-test		**	**		**	**	**	**	**	**	**	**	**

^†^ F-test for significant differences between experimental sites. * *p* < 0.05, ** *p* < 0.01.

**Table 2:** Descriptive statistics of the performance of Zn-enhanced testcross hybrids grown under optimum, low N and managed drought conditions.

Environments	Management	Statistics	Traits ^Ұ^					
			AD	ASI	PH	GY	Zn	Fe
CIMMYT 2018/19 (E1)	Optimum	Mean	69.2	1.40	218	6.80	30.7	29.7
		Range	60.0–74.0	−1.00–3.00	163–253	2.90–13.7	12.9–46.7	11.3–55.1
		SD	2.16	0.74	20.8	2.10	6.39	8.14
ART farm 2018/19 (E2)	Optimum	Mean	68.6	1.40	245	7.50	29.9	30.1
		Range	58.0–74.0	−3.00–5.00	159–288	1.90–18.8	16.4–49.2	12.2–54.9
		SD	3.06	1.09	22.3	3.53	5.80	7.91
DR&SS 2018/19 (E3)	Low N	Mean	71.0	3.00	186	1.80	24.8	20.4
		Range	63.0–76.0	0.00–6.00	148–226	0.40–4.10	13.9–39.5	7.81–34.9
		SD	2.81	1.65	17.4	0.86	5.24	6.08
CHISUMBANJE 2018/19 (E4)	Managed Drought	Mean	68.0	2.70	174	1.80	26.2	23.0
		Range	64.0–73.0	0.00–7.00	140–205	0.40–4.10	10.7–39.4	7.10–37.6
		SD	1.88	1.50	14.1	0.82	5.09	4.85
CIMMYT 2019/20 (E5)	Optimum	Mean	69.6	1.40	207	8.50	30.5	29.9
		Range	62.0–76.0	−5.00–4.00	150–245	4.30–14.3	17.3–45.3	12.6–58.4
		SD	3.14	0.90	22.3	2.07	5.87	8.06
ART farm 2019/20 (E6)	Optimum	Mean	72.6	2.00	206	9.30	30.4	30.8
		Range	65.0–84.0	−5.00–6.00	150–240	6.50–13.0	15.6–57.8	12.5–54.4
		SD	3.75	1.28	18.8	1.30	6.32	7.69
DR&SS 2019/20 (E7)	Low N	Mean	71.1	2.90	182	2.00	25.2	23.1
		Range	64.0–76.0	0.00–7.00	145–220	0.40–5.60	12.4–36.6	9.20–37.1
		SD	2.45	1.56	16.4	0.89	4.92	6.12
CHISUMBANJE 2019/20 (E8)	Managed Drought	Mean	69.4	2.8.0	161	1.10	25.1	21.1
		Range	64.0–77.0	0.00–6.00	116–214	0.10–3.80	12.1–36.8	7.90–42.8
		SD	2.71	1.51	18.1	0.72	5.23	6.10

^Ұ^ AD, Days to 50% anthesis; ASI, Anthesis silking interval (days); PH, Plant height (cm); GY, Grain yield (t ha^−1^); Zn, grain zinc concentration (mg kg^−1^); Fe, Grain iron concentration (mg kg^−1^), SD, Standard deviation.

**Table 3:** Genotypic variance (δ^2^_g_), standard error (SE) and broad-sense heritability (H^2^) for agronomic and micronutrient traits at eight experimental sites.

Trait ^Ұ^	CIMMYT 2018/19 (E1)			ART Farm 2018/19 (E2)			DR&SS 2018/19 (E3)			Chisumbanje 2018/19 (E4)		
	δ^2^_g_	SE	H^2^	δ^2^_g_	SE	H^2^	δ^2^_g_	SE	H^2^	δ^2^_g_	SE	H^2^
AD	3.05 **	0.17	0.79	1.79	0.24	0.55	6.75 **	0.22	0.92	1.89 **	0.15	0.72
ASI	0.19 **	0.06	0.51	0.16	0.08	0.53	2.07 **	0.13	0.86	1.46 **	0.12	0.78
PH	20.4 **	1.61	0.68	305 **	1.72	0.79	202 *	1.35	0.81	83.2 **	1.09	0.65
GY	3.14 **	0.16	0.85	9.57 **	0.27	0.87	0.47 **	0.07	0.78	0.38 **	0.06	0.73
Zn	38.9 **	0.49	0.97	27.9 **	0.45	0.89	24.5 **	0.40	0.94	22.8 **	0.39	0.94
Fe	49.8 **	0.63	0.87	58.3 **	0.61	0.97	34.6 **	0.47	0.96	20.6 **	0.37	0.94
Trait	CIMMYT 2019/20 (E5)			ART Farm 2019/20 (E6)			DR&SS 2019/20 (E7)			Chisumbanje 2019/20 (E8)		
	δ^2^_g_	SE	H^2^	δ^2^_g_	SE	H^2^	δ^2^_g_	SE	H^2^	δ^2^_g_	SE	H^2^
AD	7.72 **	0.24	0.90	11.5 *	0.29	0.92	3.83 *	0.19	0.78	5.98 **	0.21	0.89
ASI	0.24 *	0.25	0.56	0.15 ^ns^	0.10	0.51	1.41 *	0.12	0.76	1.27 **	0.12	0.73
PH	274 **	1.72	0.73	217 **	1.45	0.76	142 **	1.27	0.69	86.4 *	1.39	0.63
GY	3.59 **	0.16	0.92	1.19 **	0.10	0.83	0.47 **	0.07	0.75	0.29 **	0.06	0.73
Zn	32.0 **	0.45	0.96	34.7 **	0.49	0.93	21.3 **	0.38	0.93	23.4 **	0.40	0.92
Fe	59.8 **	0.62	0.96	52.9 **	0.59	0.94	35.4 **	0.47	0.97	34.1 **	0.47	0.95

^Ұ^ AD, Days to 50% anthesis; ASI, Anthesis silking interval (days); PH, Plant height (cm); GY, Grain yield (t ha^−1^); Zn, grain zinc concentration (mg kg^−1^); Fe, Grain iron concentration (mg kg^−1^). * *p* < 0.05, ** *p* < 0.01; ns, not significant at *p* < 0.05.

**Table 4:** Genotypic variance (δ^2^_g_), Genotype × Year (δ^2^_gy_), Genotype × Environment (δ^2^_ge_), Genotype × Year × Environment (δ^2^_gye_) interaction, standard error (SE) and broad-sense (H^2^) heritability for traits of Zn-enhanced hybrids.

Trait ^Ұ^	Combined Data								
	δ^2^_g_	SE	δ^2^_gy_	SE	δ^2^_ge_	SE	δ^2^_gye_	SE	H^2^
AD	0.94 **	0.29	0.12	0.25	1.63 *	0.41	2.59 *	0.21	0.59
ASI	0.08 *	0.04	0.09	0.04	0.01	0.11	0.87 *	0.15	0.56
PH	64.0 **	13.8	11.0	7.7	41.3	20.0	174.7 **	0.42	0.69
GY	0.30 **	0.11	0.01	0.10	1.23 **	0.17	0.84 *	0.17	0.89
Zn	11.2 **	2.01	1.28	0.66	14.0 **	1.31	3.99 **	0.32	0.81
Fe	9.36 **	2.24	0.55	1.49	29.3 **	2.40	5.35 **	0.05	0.66

^Ұ^ AD, Days to 50% anthesis; ASI, Anthesis silking interval (days); PH, Plant height (cm); GY, Grain yield (t ha^−1^); Zn, grain zinc concentration (mg kg^−1^); Fe, grain iron concentration (mg kg^−1^). * *p* < 0.05, ** *p* < 0.01.

**Table 5:** Correlation of grain Zn and Fe concentrations with agronomic traits in Zn-enhanced hybrids.

Traits ^Ұ^	CIMMYT 2018/19 (E1)			Art Farm 2018/19 (E2)			DR&SS 2018/19 (E3)			Chisumbanje 2018/19 (E4)		
	GY	Zn	Fe	GY	Zn	Fe	GY	Zn	Fe	GY	Zn	Fe
AD	0.42 **	0.07	0.22	0.18 **	0.31 **	0.25	−0.72 **	0.11	−0.01	−0.48 **	0.20	−0.03
ASI	0.50 **	−0.02	0.29 *	−0.35 **	0.14	0.38 **	−0.88 **	0.08	−0.00	−0.43 **	0.04	0.15
PH	0.58 **	−0.14	−0.16	0.66 **	−0.04	−0.19	0.57 **	−0.11	−0.22 *	0.28 *	−0.30 *	−0.11
GY	–	−0.01	0.04	–	−0.12	−0.18	–	−0.00	0.06	–	−0.18	−0.12
Fe	–	0.75 **	–	–	0.72 **	–	–	0.52 **	–	–	0.67 **	–
Traits	CIMMYT 2019/20 (E5)			Art farm 2019/20 (E6)			DR&SS 2019/20 (E7)			Chisumbanje 2019/20 (E8)		
	GY	Zn	Fe	GY	Zn	Fe	GY	Zn	Fe	GY	Zn	Fe
AD	0.41 **	0.05	−0.09	−0.21	0.14	−0.02	−0.52 **	0.11	0.18	−0.32 **	0.16	0.23 *
ASI	0.16	−0.08	−0.09	−0.07	0.06	0.24	−0.64 **	0.08	0.19	−0.67 **	−0.16	−0.07
PH	0.74 **	−0.25 *	−0.29 *	0.58 **	−0.01	−0.06	0.61 **	−0.09	0.04	0.83 **	−0.11	0.02
GY	–	−0.01	−0.14	–	−0.14	−0.16	–	0.05	0.03	–	0.11	0.12
Fe	–	0.56 **	–	–	0.70 **	–	–	0.54 **	–	–	0.51 **	–
Across environments												
			Traits	GY	Zn	Fe						
			AD	−0.09	0.43 **	0.25 *						
			ASI	−0.57 **	0.34 **	0.60 **						
			PH	0.99 **	−0.39 **	−0.51 **						
			GY	–	−0.44 **	−0.43 **						
			Fe	–	0.97 **	–						

^Ұ^ AD, Days to 50% anthesis; ASI, Anthesis silking interval (days); PH, Plant height (cm); GY, Grain yield (t ha^−1^); Zn, grain zinc concentration (mg kg^−1^); Fe, Grain iron concentration (mg kg^−1^). * *p* < 0.05, ** *p* < 0.01.

**Table 6:** Description of the selected plant materials used for making Zn-enhanced testcrosses and checks.

NO.	Code	Parent Type	Nutritional Type	Origin	Zn (mg kg^−1^)	Fe (mg kg^−1^)
1	D2	Male	Zinc donor	CIMMYT-Mexico	33.85	28.27
2	D3	Male	Zinc donor	CIMMYT-Mexico	33.72	28.98
3	D5	Male	Zinc donor	CIMMYT-Mexico	30.36	35.00
4	D6	Male	Zinc donor	CIMMYT-Mexico	28.68	31.52
5	D7	Male	Zinc donor	CIMMYT-Mexico	32.18	28.06
6	D8	Male	Zinc donor	IITA	30.52	34.40
7	D9	Male	Zinc donor	IITA	30.25	26.17
8	D10	Male	Zinc donor	IITA	34.29	28.77
9	D11	Male	Zinc donor	IITA	30.09	32.14
10	D12	Male	Zinc donor	IITA	30.02	26.72
11	D13	Male	Zinc donor	IITA	27.25	28.39
12	NML1	Female	Normal	CIMMYT-SARO	34.39	34.39
13	NML3	Female	Normal	CIMMYT-SARO	28.34	28.34
14	NML5	Female	Normal	CIMMYT-SARO	30.11	30.11
15	PROA1	Female	Provitamin A	CIMMYT-SARO	30.82	28.78
16	PROA3	Female	Provitamin A	CIMMYT-SARO	28.10	34.53
17	QPM4	Female	QPM	CIMMYT-SARO	35.48	33.65
18	QPM6	Female	QPM	CIMMYT-SARO	29.19	30.67
19	C1	Check	Provitamin A	Seed company	ND	ND
20	C2	Check	Provitamin A	Seed company	ND	ND
21	C3	Check	QPM	Seed company	ND	ND
22	C4	Check	Normal	Seed company	ND	ND
23	C5	Check	Normal	Seed company	ND	ND
24	C6	Check	Normal	Seed company	ND	ND
25	C7	Check	Normal	Seed company	ND	ND

ND—Not yet determined; CIMMYT-SARO = CIMMYT Southern Africa; IITA = International Institute of Tropical Agriculture.

**Table 7:** Description of experimental sites, planting dates and crop management used.

Site/Year	Agro-Ecology	Latitude	Longitude	Altitude (masl)	Annual Rainfall (mm)	Management	Entries	Planting Time	Soil Type
CIMMYT 2018/19 (E1)	IIa	17°48′ S	31°03′ E	1483	850	Optimum	84 × 2 reps	November	Ferralsol
ART farm 2018/19 (E2)	IIa	17°42′ S	31°5′ E	1556	850	Optimum	84 × 2 reps	November	Lixisol
DR&SS 2018/19 (E3)	IIa	17°13′ S	31°03′ E	1506	850	Low N	84 × 2 reps	November	Ferralsol
Chisumbanje 2018/19 (E4)	V	20°47′ S	32°13′ E	480	450	Managed drought	84 × 2 reps	May	Vertisol
CIMMYT 2019/20 (E5)	IIa	17°48′ S	31°03′ E	1483	850	Optimum	84 × 2 reps	November	Ferralsol
ART farm 2019/20 (E6)	IIa	17°42′ S	31° 5′ E	1556	850	Optimum	84 × 2 reps	November	Lixisol
DR&SS 2019/20 (E7)	IIa	17°13′ S	31°03′ E	1506	850	Low N	84 × 2 reps	November	Ferralsol
Chisumbanje 2019/20 (E8)	V	20°47′ S	32°13′ E	480	450	Managed drought	84 × 2 reps	May	Vertisol

Masl = metres above sea level; low N = managed low nitrogen conditions.

## Data Availability

Data presented in this study are available on request from the corresponding author.
